# Molecular characterisation of *Coxiella burnetii* dairy cattle strains in Estonia

**DOI:** 10.3389/fvets.2025.1568226

**Published:** 2025-05-09

**Authors:** Kädi Neare, Lea Tummeleht, Tomas Jinnerot, Brian Lassen, Arvo Viltrop

**Affiliations:** ^1^Institute of Veterinary Medicine and Animal Sciences, Estonian University of Life Sciences, Tartu, Estonia; ^2^Department of Microbiology, Swedish Veterinary Agency, Uppsala, Sweden; ^3^Research Group for Foodborne Pathogens and Epidemiology, National Food Institute, Technical University of Denmark, Kongens Lyngby, Denmark

**Keywords:** Q fever, molecular epidemiology, domestic ruminants, PCR, phylogenetic analysis

## Abstract

*Coxiella burnetii* (*C. burnetii*), an obligate intracellular zoonotic bacterium, causes abortions, stillbirths, and birth of premature and weak offspring in animals. Sheep and goats, are considered important reservoirs of infection for humans. In Estonia, *C. burnetii* is detected serologically in domestic ruminants with the prevalence being significantly higher in dairy cattle herds than that in beef cattle herds and sheep flocks. Furthermore, *C. burnetii* DNA has previously been identified in dairy cattle. This study explored the genetic characteristics of *C. burnetii* strains to identify possible sources of the disease. The strains found in the Estonian dairy herds were examined using 15-locus multiple-locus variable number tandem repeat analysis (MLVA). Across the two herds, one complete and two partial profiles with different numbers of repeats at the studied loci were identified. A comparative analysis using the unweighted pair group method with arithmetic mean (UPGMA) and randomly selected European profiles detected two larger phylogenetic clusters associated with cattle and small ruminant species, respectively. Additionally, it revealed a relationship of Estonian profiles to *C. burnetii* profiles detected in abortion material and milk from Belgian cattle. These results provide primary genetic information regarding the *Coxiella* strains circulating in this region and indicate *C. burnetii*-induced reproductive disorders in Estonian dairy cattle herds.

## 1 Introduction

*Coxiella burnetii* (*C. burnetii*), an obligate intracellular bacterium that causes the zoonotic disease Q fever (QF), is prevalent in most regions worldwide (1, 2). The range of hosts that transmit the bacterium is broad, including birds, mammals, and humans (1, 3). Pets [cats and dogs; (4–6)], reptiles (7) and wild animals (8–10) in contact with the pathogen may act as reservoirs and spread the infection while domestic ruminants are the main source of QF for humans (4, 11). *Coxiella burnetii* has been detected in secretions, such as the amniotic fluid, milk, urine, and faeces of infected mammals (12).

The clinical signs of coxiellosis in animals vary, and the disease course can be asymptomatic. In cattle, sheep, and goats, this disease mainly causes reproductive issues, including abortions, stillbirths, and weaker offspring (1, 13). Humans are usually infected via direct contact with, or inhalation of aerosols containing *C. burnetii* present in the environment. The infection typically results in flu-like illness that manifests as fever, chills, headache, and muscle pain (1, 14). Occupation involving close contact with animals is often a major risk factor for *C. burnetii* infection (2, 11, 15, 16).

Clinical manifestations are related to the virulence of the *C. burnetii* strain (17), which depends on factors such as variations in Dot/Icm type IV secretion (18, 19) and the lipopolysaccharide structure of the strain (17, 20). Highly virulent strains are associated with greater risk in humans, often causing severe and acute forms of QF, which can lead to persistent infections (21). The potential for these strains to spread in the environment and cause outbreaks is a major concern (4, 21). Given that virulence varies across specific strains, genotyping is crucial to identify those strains that serve as a potential source of infection and pose a risk to humans (22, 23). The largest QF outbreak occurred in the Netherlands in 2008–2010, and had significant short- and long-termed physical and psychological impact on human health (24).

Genotyping of strains found in QF outbreaks revealed that the *C. burnetii* profiles that cause human illness are often genetically related to the strains found in goats (4, 25). This suggests that goat-derived strains of *C. burnetii* may be attuned to infecting humans (4, 25, 26). Certain *C. burnetii* strains isolated from goats carry unique genetic markers that may be related to increased survival and replication within the host cells, evasion of host immune responses, or enhanced transmission capabilities (25, 27, 28). The virulence of *C. burnetii* infections in humans may also be influenced by evolutionary adaptations resulting from interactions between the pathogen and goat organism (4). Additionally, the environmental conditions under which pathogen strains survive and can be transmitted (e.g., through aerosolised particles) can further contribute to the virulence factors (21).

Multi-locus variable number of tandem repeat analysis (MLVA) is a method based on polymorphisms in the number of tandem repeats at multiple specific loci within the genome. By measuring the number and size of the repeats, MLVA generates a unique genetic fingerprint for each bacterial strain. This enables the linkage of strains, facilitating outbreak source identification and providing insights into the genetic diversity of circulating strains, potential reservoirs, transmission routes, and the primary host species (22, 23).

MLVA is highly discriminative, and the inherent instability of tandem repeats can complicate the interpretation of results and lead to an overestimation of genotypic diversity by showing variations in MLVA genotypes from identical backgrounds (23, 29). To address these limitations, additional molecular methods, such as multispacer sequence (MST) and single nucleotide polymorphism (SNP) typing, are often used in studies employing MLVA to achieve higher resolution in genetic fingerprinting (23, 28, 30). Combining analytical methods enhances the detection of different *C. burnetii* subgroups and lineages with better accuracy, leading to more accurate comparisons (25, 27).

To date, substantial amount of molecular data on circulating *C. burnetii* strains are available. The open-access Microbes Genotyping database (31) facilitates *C. burnetii* molecular characterisation and typing. At the time of accession (12 April 2024), this database included information on 445 *C. burnetii* genotyping results from all continents. MLVA genotyping results suggest a common group of European *C. burnetii* MLVA genotypes with sporadic emergence of new genotypes (1, 25).

In Estonia, *C. burnetii* infections have been documented serologically in cattle, sheep, and humans (16, 32, 33). *C. burnetii* seroprevalence is significantly higher in dairy cattle and humans who have direct contact with them (16, 33).

This study aims to genetically characterise and compare *C. burnetii* strains isolated from milk samples of infected dairy cattle to examine the role of *C. burnetii* infections in cattle as a potential source of infection in humans.

## 2 Materials and methods

### 2.1 Sample collection

Five dairy cattle herds were randomly selected for individual sampling from 88 herds that had tested positive for *C. burnetii* antibodies in tank milk samples between 2013 and 2014 (33). From the selected herds, 50 mL of individual milk was collected into 60-mL plastic sampling container from 38 to 70 milking cows as a convenience sample and transported under refrigeration to the laboratory.

In the laboratory, the samples were thoroughly vortexed, and 1 mL of milk was transferred into a 1.5 mL Safe-Lock Eppendorf tube (Eppendorf AG, Hamburg, Germany), coded to anonymise the analysis and stored at −20°C until further analysis.

### 2.2 Antibody detection

To increase the probability of finding animals shedding *C. burnetii* in their milk, individual milk samples were tested for the presence of *C. burnetii* antibodies using an indirect enzyme-linked immunosorbent assay PrioCHECK Ruminant Q Fever Ab Plate Kit (Thermo Fisher Scientific, Waltham, MA, United States), according to the manufacturer’s guidelines.

### 2.3 DNA extraction and PCR analysis

DNA was extracted from seropositive milk samples using Chelex 100 resin (Bio-Rad Laboratories, Hercules, CA, United States). The purified DNA was used for subsequent molecular analyses. The *C. burnetii* DNA was detected by amplifying the *C. burnetii*-specific IS*1111* repetitive element. The DNA amplification protocol was previously described by Vaidya et al. (34), and the primers were described by Berri, Laroucau, and Rodolakis (35) (Supplementary Table 1). The positive control was purchased from VirCell Microbiologists (Granada, Spain). The amount of *C. burnetii* DNA in PCR-positive milk samples was estimated by real-time PCR using the primers and the probe as described by Boskani, Edvinsson, and Wahab (36) (Supplementary Table 1). The reaction mix consisted of PerfeCTa qPCR Toughmix (Quantabio, Beverly, MA, United States), 500 nM of each primer, 100 nM of probe, and 2 μL of DNA template in a total volume of 15 μL. Amplification was performed using ABI 7500 Fast real-time PCR platform (Thermo Fisher Scientific, Waltham, MA, United States) with the following temperature profile: 95°C for 3 min and 45 cycles at 95°C for 3 s, and 60°C for 30 s. DNA from the Nine Mile reference strain was used as the positive control. Based on previous experiments (personal communication, Tomas Jinnerot and Robert Söderlund, 2014), samples with C_t_-values <33 were considered to contain sufficient *C. burnetii* DNA for MLVA (Supplementary Table 2).

### 2.4 Genotyping by MLVA

MLVA was performed on DNA extracted from milk using 15 variable-number tandem repeat (VNTR) loci, as previously described (Supplementary Table 3) (30). Two markers (VNTR loci No. 7 and No. 12) were excluded because they produced excessively large products that could not be efficiently run in the capillary electrophoresis with the other products. The PCR amplification followed the protocol described by Arricau-Bouvery et al. (30) with the following modifications: The PCR reaction mix comprised Accustart PCR Supermix (QuantaBio, Beverly, MA, United States), 500 nM of each primer, and 1 μL DNA in a total volume of 25 μL. The following temperature profile was used for amplification: 94°C for 10 min, 40 cycles of [94°C for 30 s, 60°C for 30 s, 72°C for 30 s], and a final extension at 72°C for 5 min. Forward primers were labelled with FAM, HEX, or NED to facilitate fragment analysis. ROX-labelled GeneFlo™625 DNA ladder (Eurx, Gdańsk, Poland) was used to define the fragment sizes. Amplicons were analysed by capillary electrophoresis on an ABI PRISM 3100^®^ genetic analyser (Thermo Fisher Scientific, Waltham, MA, United States), and the fragment sizes were determined using GeneMapper Software v5.0 (Thermo Fisher Scientific, Waltham, MA, United States). The number of repeats at each locus was determined by extrapolating the sizes of the obtained amplicons, and the Nine Mile strain with a known MLVA pattern (30, 31) was used as the reference.

Laboratory analyses of collected samples (testing for the presence of *C. burnetii* antibodies, DNA extraction and PCR, and genotyping) were conducted in 2014.

### 2.5 Data analysis

Two unweighted pair group analyses with arithmetic mean (UPGMA) were performed, and a double tree was constructed to place the detected *C. burnetii* profiles in a known context, assess the relationships between different *C. burnetii* profiles (UPGMA tree A), cross-validate clustering results, and enhance analysis reliability (UPGMA tree B).

Both analyses included a Nine Mile reference profile, two profiles detected in Estonia and 67 complete and partial *C. burnetii* profiles, primarily of European origin. Randomly selected profiles (Supplementary Table 4) were obtained from previously published literature using random numbers in selection process generated with random number generator in EpiTools (37). Corrections to the number of repeats per locus were applied as needed, based on updates in the public Microbes Genotyping MLVA database (31).

The UPGMA tree A incorporated data for all the studied VNTR loci. UPGMA tree B included data for six loci that were expected to display greater diversity, according to previous studies (loci no. 23, 24, 27, 28, 33, and 34) (23, 30, 38).

Free software R version 4.3.1 “Beagle Scouts” packages ‘dendextend’ and ‘ape’ (R Core Team, Vienna, Austria) were used for UPGMAs and tree construction.

To evaluate the discriminatory power of the VNTR loci (39), Simpson’s Diversity Index (SDI) (40), also known as the Hunter-Gaston Diversity Index (HGDI) (39) in molecular studies, was calculated using the free-to-use online tool Comparing Partitions (41).

## 3 Results

In total, 318 milk samples were collected from five dairy cattle herds. Anti-*C. burnetii* antibodies were detected in 23.9% (*n* = 76) of samples. Among the seropositive samples, *C. burnetii* DNA was identified in 10 seropositive individual milk samples from four dairy cattle herds (Supplementary Table 2; Supplementary Figure 1), resulting in 4.5–24.0% *C. burnetii* DNA-positive samples among the seropositive samples per herd.

Real-time PCR analysis revealed that three samples originating from two of the herds contained sufficient amounts of *C. burnetii* DNA (C_t_ < 33; Supplementary Table 2) for performing MLVA.

Results of the MLVA of the three *C. burnetii* strains from Estonian cattle are presented in Table 1. A complete MLVA profile with repetitive alleles detected at all 15 loci was obtained for one strain (EE23). Two strains (EE31 and EE48) yielded incomplete MLVA profiles, with missing information at eight and four loci, respectively. The fragment lengths linked to loci are provided in Supplementary Table 5. Further comparison revealed differences in the number of repeats across multiple loci of the obtained profiles and the reference strain.

**Table 1:** Results of the multilocus variable-number tandem repeat analysis (MLVA) for detecting *Coxiella burnetii* strains in individual milk samples of three dairy cows from two herds.

ID^1^	VNTR locus^2^ (number of repeats)														
	01	03	20	21	22	26	30	36	23	24	27	28	31	33	34
NM^3^	4	7	15	6	6	4	6	4	9	27	4	6	5	9	5
EE23*	3	6	2.5	6	6	3	6	4	5	15	2	6	3	8	13
EE31*	−3	−8	N/A^4^	−5	N/A	N/A	6	N/A	5	−11	N/A	N/A	−23	N/A	N/A
EE48	3	N/A	N/A	6	6	4	6	4	N/A	13	N/A	7	3	9	9

1 ID, sample identification.

2 VNTR locus, variable-number tandem repeat locus.

3 NM, Nine Mile/reference strain.

4 N/A, not applicable because of missing information.

*Profiles identified in the same herd (Supplementary Table 2).

**Figure 1:**
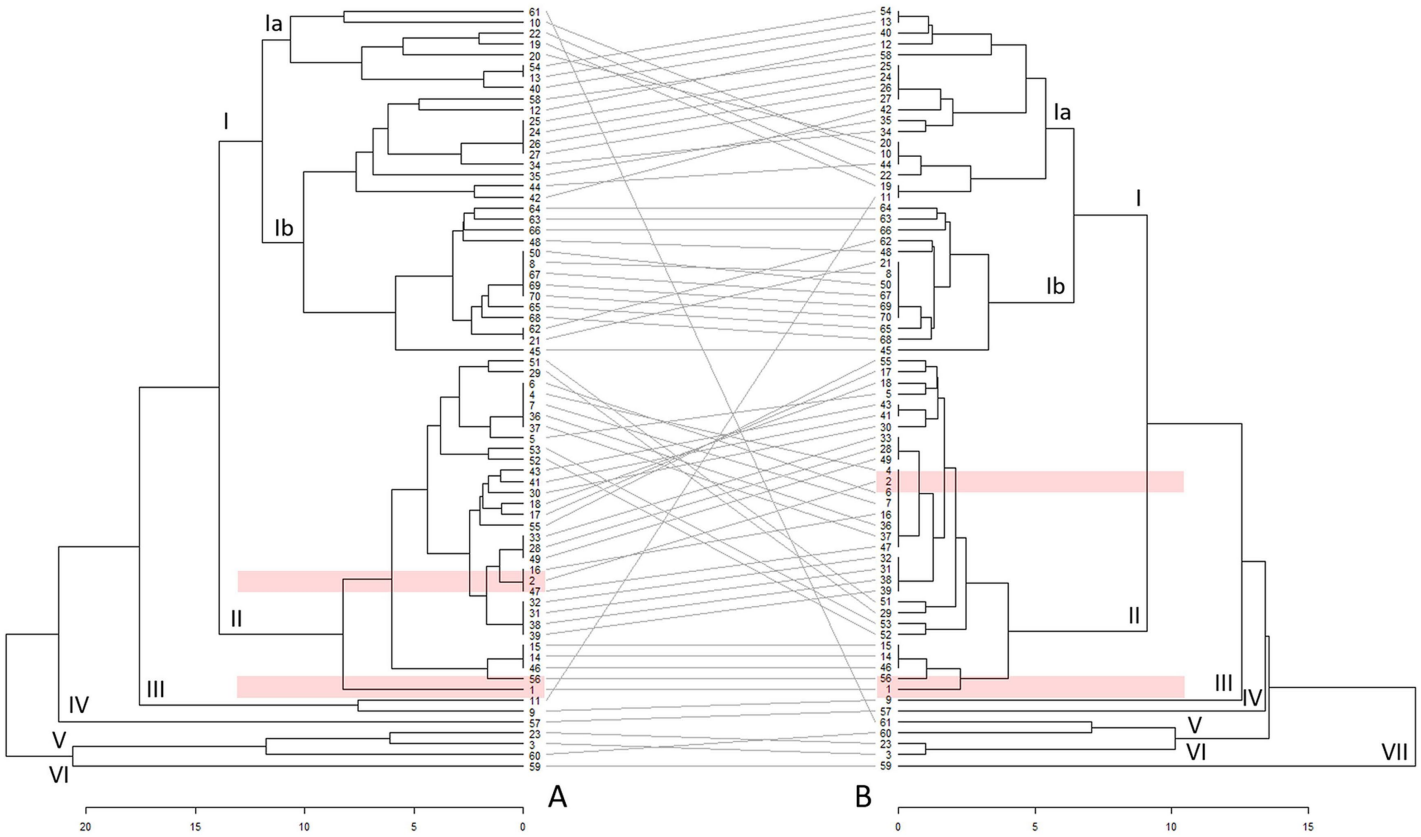
Unweighted pair group analysis with arithmetic mean (UPGMA) double tree of Estonian [marked in pink: 1 (EE23) and 2 (EE48)] and other (*n* = 68) *Coxiella burnetii* profiles based on 15 (tree **A**) and 6 (tree **B**) variable-number tandem repeat (VNTR) loci. Different clusters are marked with Roman numerals, and arrangement of profiles between the trees with grey lines. The profile numbering and origin are available in the Supplementary Table 4.

The results of the UPGMAs of the included profiles are presented in Figure 1. To depict the heterogeneity of included strains via MLVA, two minimum spanning trees were drawn (Supplementary Figure 2).

The analyses revealed six clusters at a distance of 14 in UPGMA tree A and seven clusters at a distance of 9 in UPGMA tree B (Figure 1). Cluster I represented the largest cluster in both trees. In addition, another larger cluster (cluster II) and four to five clusters, including 1–3 profiles (clusters III–VI in tree A and clusters III–VII in tree B), were identified at distances of 14 and 9, respectively. These latter profiles originated from Spain, Italy, Belgium, Poland, and the United States. The Nine Mile reference profile (Figure 1, profile No. 3) was positioned separately from the larger clusters and showed closer linkage to profiles obtained from humans in Poland and wild rabbits in Spain.

In both trees, the majority of profiles in cluster I were obtained from small ruminants and humans, whereas cluster II predominantly comprised profiles from cattle. Cluster I can be further divided into sub-clusters Ia and Ib. Sub-cluster Ia in UPGMA tree A included profiles detected in small ruminants, whereas sub-cluster Ib profiles consisted predominantly of profiles associated with humans during the Dutch QF outbreak. In the UPGMA tree B, subcluster Ia showed a mix of profiles, predominantly from small ruminants with some profiles from cattle, whereas subcluster Ib comprised mostly profiles of human origin.

Among the local strains, profiles EE23 and EE48 contained sufficient data for inclusion in UPGMA, whereas EE31 lacked information for this analysis. The two *C. burnetii* profiles found in the Estonian dairy cattle were located in cluster II in both trees. The profile of strain EE23 (Figure 1, profile No. 1) was placed separately in UPGMA tree A, whereas in tree B, the location of the profile was similar to *C. burnetii* profiles found in Italian, Greek, and French cattle herds. The strain EE48 profile (Figure 1, profile No. 2), which was associated with two profiles in UPGMA tree A, clustered into a larger group with profiles identified in cattle in Belgium, France, Croatia, and Greece.

The SDI values calculated for the 15 studied loci were based on the genotyping profiles of 70 samples included in the UPGMA (Supplementary Table 6). Values per locus ranged from 0.483 to 0.881. VNTR loci No. 34, 23, and 24 with SDI values ˃0.8 were the most informative in this study. In contrast, the VNTR locus No. 20, with an index value of <0.5, was the least diverse.

## 4 Discussion

Consistent with previous studies (33, 42, 43), *C. burnetii* DNA was isolated from the milk of cattle, indicating that the bacteria can be present in dairy cattle, underscoring that cattle can serve as a potential risk of human QF infection. Outbreaks of human QF have previously been associated with dairy cattle farming (15, 44). Because the samples studied were exclusively obtained from seropositive milk samples from seropositive dairy cattle herds and considering the intermittent *C. burnetii* shedding pattern of infected animals, the actual prevalence of *C. burnetii*-excreting cows is likely underestimated (42, 45). Asymptomatic animals may shed low levels of the pathogen in their milk (42).

Because of the differences in applied methodologies for detecting *C. burnetii* in diverse populations and the lack of harmonised data reporting, comparison of studies remains challenging (46, 47). This complicates the accurate assessment of the prevalence of *C. burnetii* excretion in cattle, both in Europe and globally.

### 4.1 MLVA characterisation

This is the first study to characterise the profiles of *C. burnetii* circulating in domestic ruminants in Estonia. In recent decades, molecular characterisation has become an important tool for clarifying the genetic variability of pathogens, including *C. burnetii*, and identifying possible outbreak sources and transmission routes (1, 28).

MLVA was used to identify *C. burnetii* profiles in Estonian dairy cattle herds. This technique was selected for its direct applicability to purified DNA (30). However, the outcomes may be overly discriminative and limited in reproducibility, as MLVA relies on relatively unstable DNA partitions, and comparisons of results from capillary electrophoresis machinery can be challenging (23, 29). Therefore, MLVA results should be evaluated and compared with caution.

In this study, one complete and two partial *C. burnetii* profiles were identified in individual milk samples. However, the success of MLVA typing was diminished for samples with real-time PCR C_t_-values exceeding 33, which was attributed to the limited quantity of DNA in those samples. Consequently, certain samples could not be profiled, and a complete 15 loci MLVA profile was obtained for only one sample. For one profile (EE31), a negative number of repeats was detected in five studied loci, which indicates potential technical or interpretational bias. Because three samples were analysed and interpreted together, this may further indicate inadequate DNA quantity and quality in the sample.

Moreover, the number of detected profiles may have been influenced by the inclusion of only antibody-positive samples in the analysis. Domestic ruminants can excrete *C. burnetii* bacteria and simultaneously test negative for antibodies (48, 49). However, because *C. burnetii* DNA was found in only a few antibody-negative BTM samples (48, 49), the number of missed positive samples in this study is probably small.

The three identified *C. burnetii* profiles (two partial and one complete) exhibited heterogeneity. Profiles that differ by a few markers have been observed in other European countries (28, 38), and the three Estonian profiles likely represent microvariants of each other as well as strains found in Europe.

Despite the availability of extensive data on the profiles of *C. burnetii* circulating in Western and Central European countries, information on strains from Eastern and Northern Europe is relatively limited. Furthermore, the amount of data available worldwide varies greatly, leading to possible bias (25). This highlights the need for comprehensive research in animal and human populations and the importance of systematically depositing data into public databases. Furthermore, studies have used different profiling methods, making it beneficial to incorporate the results of different analyses to harmonise data and databases and obtain a broader overview of the molecular epidemiology of *C. burnetii* (25, 28, 50, 51). In this study, alternative profiling methods, such as whole-genome sequencing and SNP analysis, could not be used because of the small amount of DNA in the samples.

### 4.2 Data analysis

In this study, UPGMA was incorporated to place the identified profiles into a known context and assess the potential health risks of human and animal infections associated with the strains found in Estonia. However, the analysis may result in profile misplacements, as unrelated profiles may display identical numbers of tandem repeats (28). These misplacements can lead to distorted understanding of genetic diversity, underestimated zoonotic effect of the strain, and its impact on human health. Therefore, clustering results should be approached with some caution.

As one of the Estonian profiles was incomplete, UPGMA tree A was constructed using partial *C. burnetii* profiles with data from six or fewer loci from other studies. To address the limited loci in tree A, UPGMA tree B was created. The two trees were then compared to enhance the reliability of the clustering in UPGMA tree A.

Both UPGMA trees revealed the presence of two larger clusters. Similar clustering of profiles isolated from domestic ruminant hosts (25, 28, 38) and wildlife (52) has been reported in other studies, indicating host specificity. Furthermore, *C. burnetii* profiles detected in sheep and goats tend to form two separate clusters (25, 38). This trend was not as clearly observed in this study, as the number of small ruminant profiles was limited (Supplementary Table 4) because of the rarity of *C. burnetii* infection in sheep and goats in Estonia (33). Additionally, clusters of sheep and goat profiles tend to be more genetically diverse and include or are linked to profiles detected in humans (28, 38). As QF has not been diagnosed and *C. burnetii* isolated from humans in Estonia (53), the study lacks comparison with local human profiles. As an earlier study revealed higher probability for seropositivity for *C. burnetii* in people professionally involved with dairy cattle compared to other population groups in Estonia (16), the infection is likely underdiagnosed.

The heterogeneous structure within subcluster Ib in UPGMA tree A and subcluster Ia in UPGMA tree B may indicate a potentially heightened evolutionary capacity, possibly linked to an enhanced ability to infect several host species. Some level of heterogeneity has been previously reported (25, 27, 28, 38). Increased genome plasticity, mutations in membrane proteins, and predicted virulence-associated genes in some Dutch and Belgian profiles have been proposed as factors contributing to the ability to affect several host species (28, 54). Additionally, the observed occurrence of similar profiles in various animal species may indicate interspecies transmission (25, 27, 28, 38).

Unlike profiles from goats and sheep, *C. burnetii* profiles linked to cattle have exhibited less genetic variation over time. This stability may reduce the risk of large-scale human outbreaks from cattle-derived strains (27, 28, 38). At the same time, *C. burnetii* strains detected in cattle in Poland may pose a zoonotic threat, thus continuous monitoring is essential to track potential changes in their virulence or transmission patterns (15, 43). Some *C. burnetii* profiles tend to remain within specific regions, suggesting that local factors (e.g., farming practices and biosecurity measures) influence their persistence. This regional stability can help tailor disease control strategies (38, 43).

In both trees, profiles from Estonian cattle were not closely related to those currently described in humans and small ruminants but rather related to profiles found in other cattle. Profiles that closely resembled the Estonian profile EE48 (Figure 1, profile No. 2) have also been detected in bovine abortion materials from Belgium (28). The high similarity in numbers of repeats at the studied loci and the close proximity between these profiles, and *C. burnetii* identification in bovine abortion material (55) suggests that QF outbreaks in Estonian dairy cattle herds are possible. Moreover, based on personal observations and the abovementioned similarities between Estonian, Belgian, and French *C. burnetii* profiles, the transport of live animals and breeding material from Western European countries may have contributed to the spread of *C. burnetii* (56). *C. burnetii* can be transmitted through semen (57, 58) and embryos (59, 60). The stability of *C. burnetii* profiles (27, 38, 50) supports these claims. Therefore, increasing the awareness of the disease among dairy producers, healthcare practitioners, and other stakeholders is necessary.

Comparison of the constructed trees revealed differences in cluster compositions and distances between the profiles and clusters. Profile rearrangements inside and between the clusters (Figure 1, grey lines between the trees), shortening of tree branches, and distances between clusters and data points in UPGMA tree B were possible because of the larger amount of available information (28). Although the profiles used to construct UPGMA tree A contained less information about the number of tandem repeats at the included loci than UPGMA tree B, the UPGMA tree A can be considered reliable, because there are relatively few rearrangements of clusters and individual *C. burnetii* profiles when compared to UPGMA tree B.

### 4.3 Concluding remarks

The *C. burnetii* strains detected in this study exhibit similarities to European strains mainly found in cattle and associated with reproductive issues. Although these strains have not demonstrated virulence in humans, the limited sampling of herds reflects primarily the situation in studied herds, warranting further studies on *C. burnetii* molecular characterisation to adequately assess the human and animal health risks posed by QF in Estonia. Given the potential limitations of MLVA for this specific sample set, incorporating additional laboratory analysis in future studies should be considered.

## Data Availability

The data generated for this study can be found in the Microbes Genotyping MLVAbank (available at: https://microbesgenotyping.i2bc.paris-saclay.fr/databases/public) sheet “Coxiella burnetii_v3_2”. Additionally, the detected C. burnetii profiles can be found in the NCBI online repository (available at: https://www.ncbi.nlm.nih.gov/) under the following accession codes: SAMN47866579, SAMN47866580, SAMN47866581.
