# Supplementary material for: Molecular characterisation of Coxiella burnetii dairy cattle strains in Estonia
Source: Front Vet Sci. 2025 May 9;12:1568226. doi: 10.3389/fvets.2025.1568226 (PMC12098354; doi:10.3389/fvets.2025.1568226)
Supplement: Supplementary file 1 [file Table_1.docx]

**Supplementary Table 1.** Primers and the probe exploited in the detection of *Coxiella burnetii* DNA from individual dairy cattle milk samples

| **Primer/probe** | **Sequence** | **Reference** |
| --- | --- | --- |
| Trans-1 | TATGTATCCACCGTAGCCAGTC | Berri, Laroucau, and Rodolakis (2000) |
| Trans-2 | CCCAACAACACCTCCTTATTC | Berri, Laroucau, and Rodolakis (2000) |
| IS*1111*a-F | CACGAGACGGGTTAAG | Boskani, Edvinsson, and Wahab (2018) |
| IS*1111*a-R | CACACGCTTCCATCAC | Boskani, Edvinsson, and Wahab (2018) |
| IS*1111*a-P | FAM-TCAGTATGTATCCACCGTAGCCA-BHQ1 | Boskani, Edvinsson, and Wahab (2018) |

**References:**

Berri, M., K. Laroucau, and A. Rodolakis. 2000. “The Detection of Coxiella Burnetii from Ovine Genital Swabs, Milk and Fecal Samples by the Use of a Single Touchdown Polymerase Chain Reaction.” *Veterinary Microbiology* 72 (3–4): 285–93. https://doi.org/10.1016/S0378-1135(99)00178-9.

Boskani, T., B. Edvinsson, and T. Wahab. 2018. “Development of Nineteen Taqman Real-Time PCR Assays for Screening and Detection of Select Highly Pathogenic Bacteria.” *Infection Ecology & Epidemiology* 8 (1): 1553462. https://doi.org/10.1080/20008686.2018.1553462.
