# Supplementary material for: Molecular characterisation of Coxiella burnetii dairy cattle strains in Estonia
Source: Front Vet Sci. 2025 May 9;12:1568226. doi: 10.3389/fvets.2025.1568226 (PMC12098354; doi:10.3389/fvets.2025.1568226)
Supplement: Supplementary file 2 [file Table_2.docx]

**Supplementary Table 2.** Determined cycle threshold values of individual milk samples found to be positive for the presence of *Coxiella burnetii*-specific IS*1111* fragment

| **ID^1^** | **County** | **Herd ID** | **C_t_-value^2^** |
| --- | --- | --- | --- |
| EE23 | Järva | 1 | 30.19 |
| EE31 | Järva | 1 | 32.91 |
| EE40 | Järva | 2 | 36.95 |
| EE48 | Järva | 2 | 32.75 |
| EE49 | Järva | 2 | N/A^3^ |
| EE51 | Järva | 2 | N/A |
| EE52 | Järva | 2 | 35.85 |
| EE95 | Järva | 2 | 33.27 |
| EE159 | Jõgeva | 3 | 33.06 |
| EE291 | Lääne-Viru | 4 | 35.93 |

^1^ ID: sample number

^2^ C_t_-value: cycle threshold value

^3^ N/A: information not available
