# Supplementary material for: Molecular characterisation of Coxiella burnetii dairy cattle strains in Estonia
Source: Front Vet Sci. 2025 May 9;12:1568226. doi: 10.3389/fvets.2025.1568226 (PMC12098354; doi:10.3389/fvets.2025.1568226)
Supplement: Supplementary file 3 [file Table_3.docx]

**Supplementary Table 3.** Primers used in the multi-locus variable number of tandem repeat analysis (MLVA) of milk samples containing *Coxiella burnetii* DNA

| **VNTR^1^** | **Locus name^2^** | **Sequence (5’ -> 3’)^3^** | **5’ marker** |
| --- | --- | --- | --- |
| 01 | Cbu0033_ms01_16bp_4U_198bp | Fw: GGCTCATTCAATTTTAGCTTCG | HEX |
|  |  | Rv: AACGTGGGGAAGTTTGTTATTT |  |
| 03 | Cbu0448_ms03_12bp_7U_229bp | Fw: TTGTCGATAAATCGGGAAACTT | FAM |
|  |  | Rv: CACTGGGAAAAGGAGAAAAAGA |  |
| 20 | Cbu1941_ms20_18bp_15U_402bp | Fw: CTGAAACCAGTCTTCCCTCAAC | NED |
|  |  | Rv: CTTTATCTTGGCCTCGCCCTTC |  |
| 21 | Cbu1963_ms21_12bp_6U_210bp | Fw: AGCATCTGCCTTCTCAAGTTTC | HEX |
|  |  | Rv: TGGGAGGTAGAAGAAAAGATGG |  |
| 22 | Cbu1980_ms22_11bp_6U_246bp | Fw: GGGGTTTGAACATAGCAATACC | NED |
|  |  | Rv: CAATATCTCTTTCTCCCGCATT |  |
| 26 | Cbu0831_ms26_9bp_4U_127bp | Fw: AGAATCAAACCTGCAAAACCTT | NED |
|  |  | Rv: TTGATTATTTTGACTTCGCTGGT |  |
| 30 | Cbu1351_ms30_18bp_6U_306bp | Fw: ATTTCCTCGACATCAACGTCTT | FAM |
|  |  | Rv: AGTCGATTTGGAAACGGATAAA |  |
| 36 | Cbu1941_ms36_9bp_4U_477bp | Fw: GAAACCAGTCTTCCCTCAACAG | FAM |
|  |  | Rv: ATAACCGTCATCGTCACCTTCT |  |
| 23 | Cbu0197_ms23_7bp_8U_157bp | Fw: GGACAAAAATCAATAGCCCGTA | FAM |
|  |  | Rv: GAAAACAGAGTTGTGTGGCTTC |  |
| 24 | Cbu0259_ms24_7bp_27U_344bp | Fw: ATGAAGAAAGGATGGAGGGACT | HEX |
|  |  | Rv: GATAGCCTGGACAGAGGACAGT |  |
| 27 | Cbu0838_ms27_6bp_4U_276bp | Fw: TTTTGAGTAAAGGCAACCCAAT | FAM |
|  |  | Rv: CAAACGTCGCACTAACTCTACG |  |
| 28 | Cbu0839_ms28_6bp_6U_276bp | Fw: TAGCAAAGAAATGTGAGGATCG | FAM |
|  |  | Rv: ATTGAGCGAGAGAATCCGAATA |  |
| 31 | Cbu1418_ms31_7bp_5U_285bp | Fw: GGGCATCTAATCGAGATAATGG | HEX |
|  |  | Rv: TTTGAGAAAATTTTGGGTGCTT |  |
| 33 | Cbu1435_ms33_7bp_9U_262bp | Fw: TAGGCAGAGGACAGAGGACAGT | FAM |
|  |  | Rv: ATGGATTTAGCCAGCGATAAAA |  |
| 34 | Cbu1471_ms34_6bp_5U_210bp | Fw: TGACTATCAGCGACTCGAAGAA | NED |
|  |  | Rv: TCGTGCGTTAGTGTGCTTATCT |  |

^1^ VNTR: variable number tandem-repeat (VNTR) locus number

^2^ Locus name according to Arricau-Bouvery et al. (2006). Corrected loci have been used for VNTR 30, 36 and 31

^3^ Sequence: forward (Fw) and reverse (Rv) sequences according to Arricau-Bouvery et al. (2006)

**Reference:**

Arricau-Bouvery, N., Y. Hauck, A. Bejaoui, D. Frangoulidis, C. C. Bodier, A. Souriau, H. Meyer, H. K. J. Neubauer, A. Rodolakis, and Gilles Vergnaud. 2006. “Molecular Characterization of Coxiella Burnetii Isolates by Infrequent Restriction Site-PCR and MLVA Typing.” *BMC Microbiology* 6: 1–14. https://doi.org/10.1186/1471-2180-6-38.
