# Supplementary material for: Molecular characterisation of Coxiella burnetii dairy cattle strains in Estonia
Source: Front Vet Sci. 2025 May 9;12:1568226. doi: 10.3389/fvets.2025.1568226 (PMC12098354; doi:10.3389/fvets.2025.1568226)
Supplement: Supplementary file 5 [file Table_5.docx]

**Supplementary Table 5.** Fragment lengths obtained from the multi-locus variable number of tandem repeat analysis (MLVA) of individual milk samples

| **ID** | **VNTR flanking region^1^ (bp)** | | | | | | | | | | | | | | |
| --- | --- | --- | --- | --- | --- | --- | --- | --- | --- | --- | --- | --- | --- | --- | --- |
|  | **01** | **03** | **20** | **21** | **22** | **26** | **30** | **36** | **23** | **24** | **27** | **28** | **31** | **33** | **34** |
| NM^2^ | 194.25 | 228.97 | 400.74 | 209.10 | 245.64 | 123.58 | 307.73 | 475.35 | 151.86 | 342.80 | 275.82 | 277.25 | 280.77 | 260.20 | 206.89 |
| EE23 | 217.50 | 217.50 | 171.58 | 208.87 | 245.72 | 114.64 | 307.90 | 474.30 | 130.52 | 259.00 | 264.71 | 277.45 | 267.09 | 252.97 | 254.96 |
| EE31 | 75.23 | 54.59 | N/A^3^ | 75.46 | N/A | N/A | 307.70 | N/A | 131.55 | 75.40 | N/A | N/A | 88.31 | N/A | N/A |
| EE48 | 178.41 | N/A | N/A | 208.77 | 245.52 | 123.73 | 307.74 | 474.45 | N/A | 244.66 | N/A | 283.15 | 268.07 | 260.31 | 231.27 |

^1^ variable-number tandem repeat loci’s flanking regions primer lengths in base pairs

^2^ NM: Nine Mile strain/reference

^3^ N/A: no available data due to missing information
