# Supplementary material for: Molecular characterisation of Coxiella burnetii dairy cattle strains in Estonia
Source: Front Vet Sci. 2025 May 9;12:1568226. doi: 10.3389/fvets.2025.1568226 (PMC12098354; doi:10.3389/fvets.2025.1568226)
Supplement: Supplementary file 6 [file Table_6.docx]

**Supplementary Table 6.** Simpson’s Diversity Indices (SDIs) of studied loci, based on 70 included profiles

| **VNTR^1^** | **No. profiles^2^** | **SDI^3^** | **CI (95%)^4^** |
| --- | --- | --- | --- |
| 01 | 3 | 0.530 | (0.424-0.636) |
| 03 | 3 | 0.664 | (0.633-0.694) |
| 20 | 6 | 0.483 | (0.354-0.612) |
| 21 | 3 | 0.520 | (0.465-0.575) |
| 22 | 3 | 0.525 | (0.475-0.575) |
| 26 | 9 | 0.554 | (0.431-0.678) |
| 30 | 6 | 0.762 | (0.698-0.826) |
| 36 | 7 | 0.696 | (0.632-0.760) |
| 23 | 10 | 0.824 | (0.789-0.859) |
| 24 | 13 | 0.819 | (0.755-0.883) |
| 27 | 6 | 0.618 | (0.544-0.693) |
| 28 | 5 | 0.617 | (0.551-0.683) |
| 31 | 4 | 0.616 | (0.557-0.675) |
| 33 | 10 | 0.733 | (0.643-0.823) |
| 34 | 12 | 0.881 | (0.849-0.913) |

^1^ VNTR: variable-number tandem repeat locus number

^2^ No. profiles: number of profiles detected in the locus

^3^ SDI: Simpson’s Diversity Index, an index of zero presents complete uniformity

^4^ CI (95%): 95% confidence intervals
