# Supplementary material for: Molecular characterisation of Coxiella burnetii dairy cattle strains in Estonia
Source: Front Vet Sci. 2025 May 9;12:1568226. doi: 10.3389/fvets.2025.1568226 (PMC12098354; doi:10.3389/fvets.2025.1568226)
Supplement: Supplementary file 7 [file Image_1.pdf]

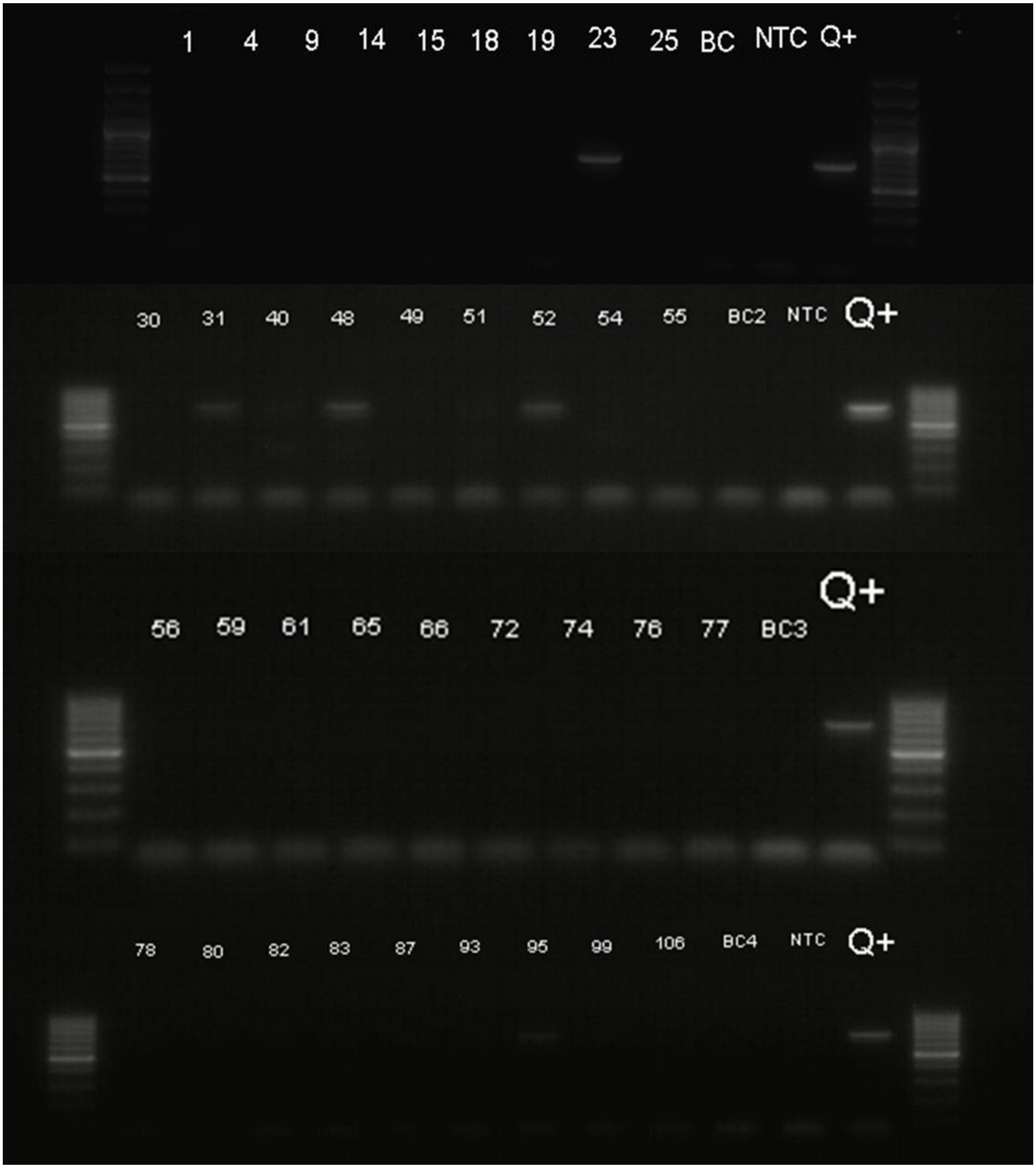
1 4 9 14 15 18 19 23 25 BC NTC Q+
30 31 40 48 49 51 52 54 55 BC2 NTC Q+
Q+
56 59 61 65 66 72 74 76 77 BC3
78 80 82 83 87 93 95 99 106 BC4 NTC Q+

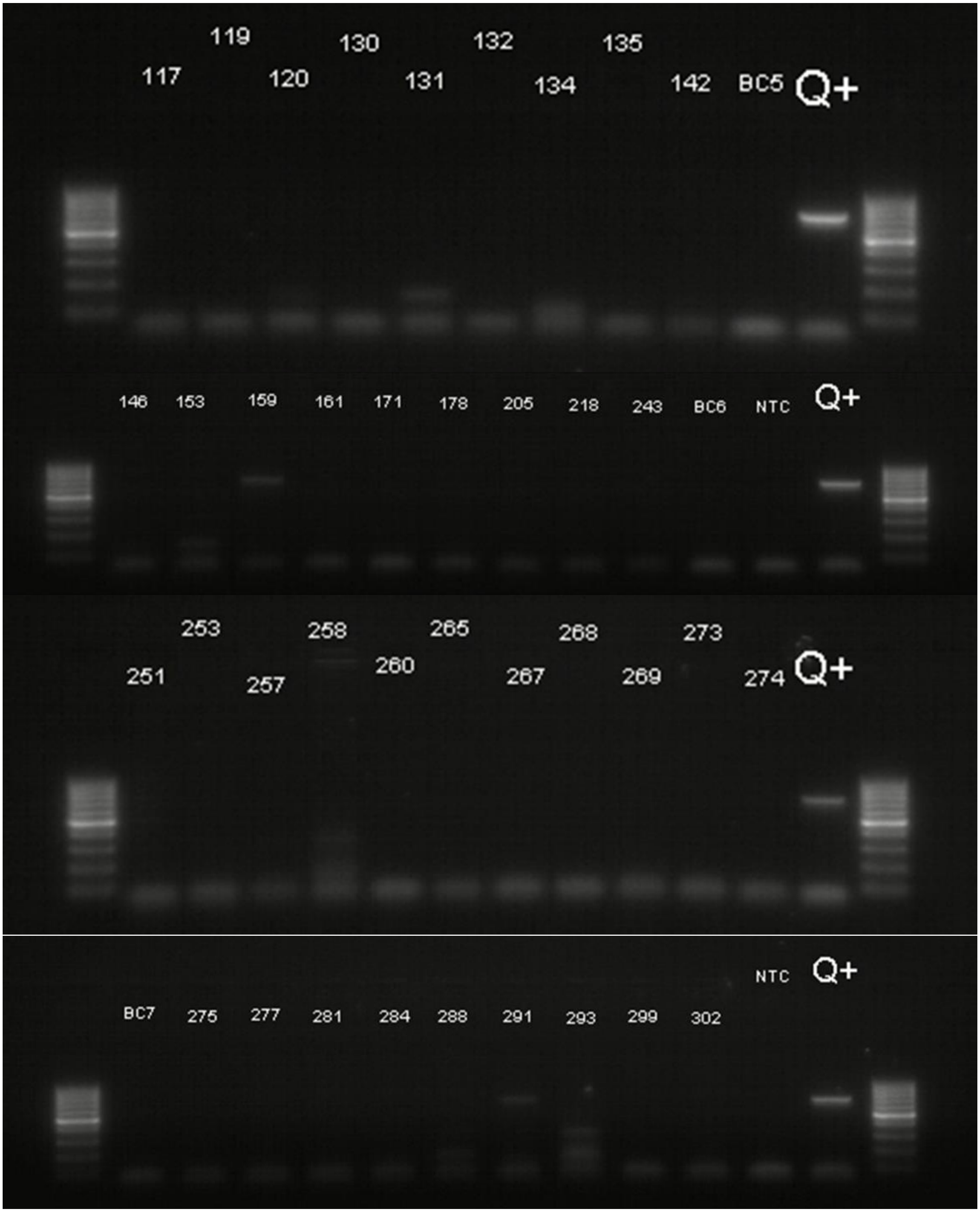

Supplementary Figure 1. Images of gels of the polymerase chain reaction (PCR) detecting *Coxiella burnetii* DNA in individual dairy cattle milk samples tested seropositive for the bacterium. NTC: no template negative control; Q+: positive control of *Coxiella burnetii* DNA; BC: blank control of DNA extraction. The figure is missing results of two samples tested negative for the *Coxiella. burnetii* DNA presence (analysed separately)
