# Supplementary material for: Molecular characterisation of Coxiella burnetii dairy cattle strains in Estonia
Source: Front Vet Sci. 2025 May 9;12:1568226. doi: 10.3389/fvets.2025.1568226 (PMC12098354; doi:10.3389/fvets.2025.1568226)
Supplement: Supplementary file 8 [file Image_2.pdf]

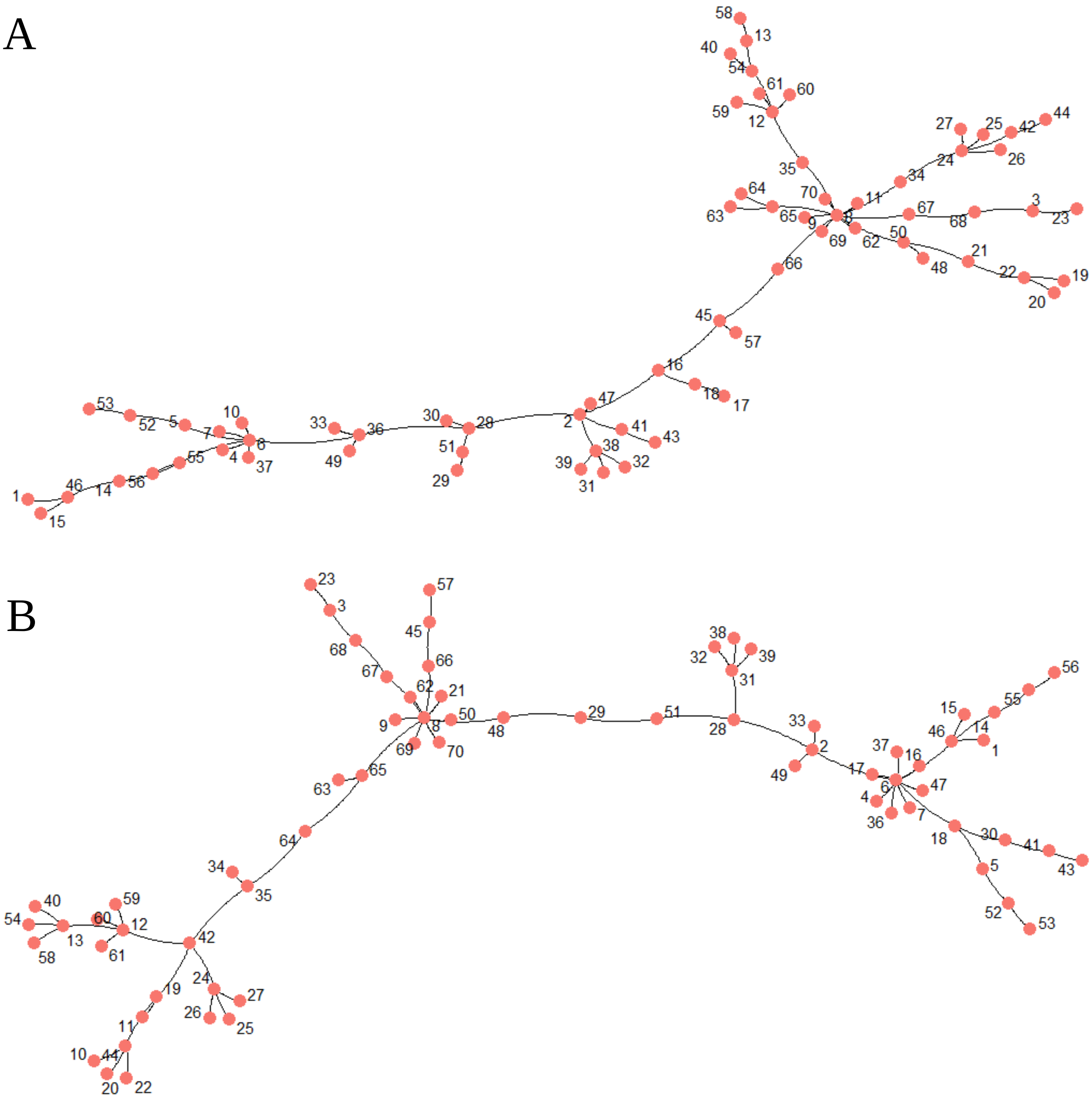

Supplementary Figure 2. Minimum spanning trees of Estonian [No. 1 (EE23) and No. 2 (EE48)] and other (n = 68) *Coxiella burnetii* profiles based on 15 (tree A) and 6 (tree B) variable-number tandem repeat (VNTR) loci. The numbering and origin of profiles is presented in the *Supplementary Table 4*
